# Neurocognitive development of novelty and error monitoring in children and adolescents

**DOI:** 10.1038/s41598-021-99043-z

**Published:** 2021-10-06

**Authors:** Kathleen Kang, Nina Alexander, Jan R. Wessel, Pauline Wimberger, Katharina Nitzsche, Clemens Kirschbaum, Shu-Chen Li

**Affiliations:** 1 Faculty of Psychology, Chair of Lifespan Developmental Neuroscience, Technische Universität Dresden, Zellescher Weg 17, BZW A232/233, 01069 Dresden, Germany; 2 Department of Psychiatry and Psychotherapy, Phillips University Marburg, Marburg, Germany; 3 Center for Mind, Brain and Behavior, Philipps University Marburg, Marburg, Germany; 4 Department of Psychological and Brain Sciences and Department of Neurology, University of Iowa, Iowa City, USA; 5 Department of Gynecology and Obstetrics, Faculty of Medicine, Technische Universität Dresden, Dresden, Germany; 6 Faculty of Psychology, Chair of Biological Psychology, Technische Universität Dresden, Dresden, Germany; 7 Centre for Tactile Internet with Human-in-the-Loop, Technische Universität Dresden, Dresden, Germany

**Keywords:** Human behaviour, Cognitive neuroscience

## Abstract

The abilities to monitor one’s actions and novel information in the environment are crucial for behavioural and cognitive control. This study investigated the development of error and novelty monitoring and their electrophysiological correlates by using a combined flanker with novelty-oddball task in children (7–12 years) and adolescents (14–18 years). Potential moderating influences of prenatal perturbation of steroid hormones on these performance monitoring processes were explored by comparing individuals who were prenatally exposed and who were not prenatally exposed to synthetic glucocorticoids (sGC). Generally, adolescents performed more accurately and faster than children. However, behavioural adaptations to error or novelty, as reflected in post-error or post-novelty slowing, showed different developmental patterns. Whereas post-novelty slowing could be observed in children and adolescents, error-related slowing was absent in children and was marginally significant in adolescents. Furthermore, the amplitude of error-related negativity was larger in adolescents, whereas the amplitude of novelty-related N2 was larger in children. These age differences suggest that processes involving top-down processing of task-relevant information (for instance, error monitoring) mature later than processes implicating bottom-up processing of salient novel stimuli (for instance, novelty monitoring). Prenatal exposure to sGC did not directly affect performance monitoring but initial findings suggest that it might alter brain-behaviour relation, especially for novelty monitoring.

## Introduction

The volatility of everyday life is often associated with experiencing unexpected or novel external events in the environment on the one hand, and with unforeseen negative outcomes resulting from personal actions on the other hand. Both stimulus novelty and action errors carry valuable information for individuals to adapt their behaviours. To act and to reach our goals in daily life, flexible and goal-directed behaviour is essential to adapt to such ever-changing interplays between external events and internal processes of action regulation. Goal-directed behaviour thus needs to rely on successful cognitive monitoring, which entails a set of neurocognitive processes that constantly examines the occurrence of undesirable personal action outcomes (error monitoring) and novel external events (novelty monitoring).

Though the errors resulting from one’s own actions are usually unforeseen and undesired, if detected, they carry valuable information that can be used to adjust the subsequent course of action and behaviour. Cognitive neuroscience research over the past three decades has consistently demonstrated evidence for successful error monitoring in the healthy adult population through a phenomenon known as ‘post-error slowing (PES)’: specifically, individuals are usually slower on trials following an error. This effect has been observed across a variety of different tasks, such as: the Flanker^1–3^, Stroop^4^ and Simon tasks^5^. Such PES has been suggested to reflect, in part, the process of a compensatory regulatory mechanism^4^ that supports more top-down controlled responses^6^ on subsequent trials. It is also related to reduced motor cortex activity^5,7^ which reflects an increased response threshold in post-error trials. The PES is often accompanied by the so-called error-related negativity (ERN), which is a negative deflection measured by electroencephalography (EEG) at the fronto-central electrodes within approximately 100 ms of an error^8,9^. Previous findings showed that the amplitude of ERN predicts PES^10,11^ and it is, therefore, considered to be a marker of processes related to error detection and error monitoring^12,13^. Although the PES and its neural correlate, ERN, have been considered as manifestations of adaptive control for subsequent actions, it has also been suggested that performance slowing after errors may, alternatively, reflect the fact that errors are unexpected, which render the PES as an indication of unanticipated process disruption^14–16^.

Turning to the topic of novelty monitoring, novel events have inherent value in changing and dynamic environments since they signal information that have not yet been encountered, remembered, or learned. Thus, apart from the work on error monitoring, past research has also investigated the cognitive process for detecting and monitoring novel events. Novelty monitoring is typically investigated using novelty-oddball paradigms^17,18^ in which participants monitor a series of stimuli for task-relevant but unusual stimuli (“oddballs”) while task-irrelevant “novel” stimuli that are unbeknownst to the participants are also occasionally presented amidst other stimuli. The occurrence of these novel stimuli often evoke motor slowing^19^, which is a behaviour effect usually termed as “post-novelty slowing” (PNS). For example, while driving along an urban road, one might slow down at the detection of a ‘novel’ stimuli in the environment, such as special signs announcing road closures due to rare sport events or unforeseen weather conditions. Such PNS is usually accompanied by the N2 component of EEG signals. Unlike the ERN, the N2 component is stimulus-locked and precedes the responses. Its amplitude is determined by the processing of irrelevant or distracting information, especially when the discrepancy between long-term exposure to recurring visual stimuli and exposure to novel stimuli produces a deviation from the expected perceptual template^17^. Therefore, both PNS and the novelty-associated N2 component have been considered as markers of performance monitoring processes^20,21^ that are related to novelty monitoring.

Although the processes of error and novelty monitoring are often studied independently, the occurrence of both types of events are usually unforeseen or unexpected and require rapid cognitive and behavioural adaptation. Due to these similarities, both ERN and the PNS-associated N2 have been shown to be generated from a similar neural substrate^22–24^, i.e., the anterior cingulate cortex (ACC) and more specifically, the mid-cingulate cortex (aMCC), that are both part of the medial prefrontal cortex (mPFC). Ample evidence suggests that activities in ACC or aMCC increase when conflicting, undesired, or unanticipated events are detected during information processing, which signal the need for increased cognitive control in these situations^6,21^. While both ERN and N2 may share a similar neural generator, it should be acknowledged that each component is sensitive to different aspects of performance monitoring; whereas the PES-associated ERN reflects the processing of task-relevant information (i.e., one’s own action error), the PNS-associated N2 reflects the processing of task-irrelevant information (i.e., non-relevant external stimuli). Given this difference, it is likely that error and novelty monitoring may show different developmental time courses during childhood and adolescence.

It is well established in the developmental literature that there is a delayed maturation of the prefrontal cortex (PFC) in children and adolescents^25,26^. Delayed maturation of the PFC may limit cognitive control processes such as performance monitoring. Indeed, a greater accuracy in monitoring stimulus–response conflict (e.g., observed with the Go/NoGo task) has been shown to be associated with stronger frontostriatal white matter connections^27^, which still undergoes the process of maturation in adolescence. At the functional level, stronger activation in the prefrontal, parietal and striatal brain regions are associated with successful behavioural inhibition in NoGo than in Go trials. Furthermore, this effect was more apparent in children than in adults, which reflects that this brain network plays an important role in the development of performance monitoring^28^. As the prefrontal cortex matures gradually during childhood and adolescence, cognitive control skills also improve with age^29,30^. According to this view, children’s ability to ‘correct’ their errors would also improve with their increasing ability to engage cognitive control skills; and this error-correcting ability is reflected in a larger ERN. Initially, children showed prolonged reaction times and have a higher tendency to commit errors in conditions involving stimulus–response conflicts^31–33^. However, ERN increases linearly with age during development and a stronger ERN was associated with increased accuracy and faster reaction times^34^. As children improve in their ability to engage their cognitive control skills, they should also improve in their ability to filter distracting or inhibiting interfering information which may subsequently lead to a reduced N2 amplitude. The frontally distributed N2 component has been shown to be larger in children than in other age groups and under high conflict conditions in the Go/NoGo task^31,35,36^, suggesting that a more extensive PFC activity is needed during response inhibition in the presence of novel stimuli. Taking these findings together, a review of earlier results^37^ and a recent meta-analysis^38^ demonstrated a clear dissociation in the effects of age on ERN and N2, with the amplitude of ERN activity increasing (stronger ERN activity) but the amplitude of N2 activity decreasing (weaker N2 activity) with age during development. Therefore, although performance monitoring generally improves with age during development, the developmental trajectories for neural correlates underlying error (ERN) and novelty (N2) monitoring of task-relevant and task-irrelevant information, respectively, may be different.

The development of performance monitoring processes can be influenced by other factors in the developmental context, such as prenatal exposure to synthetic glucocorticoids (sGC). SGCs (e.g., betamethasone or dexamethasone) are often prescribed to pregnant women who are at risk of preterm birth to prevent infant lung disease and breathing difficulties in the first few weeks of life, as well as long-term disabilities such as epilepsy and cerebral palsy^39^. Despite the benefits of sGCs in these situations, repeated antenatal exposure to sGCs can result in delayed brain maturation^40^. During prenatal development, sGCs can readily cross the placenta barrier, thus directly exposing the fetus to excessive levels of glucocorticoids (GC), which has been shown to cause dysregulation in the hypothalamic–pituitary–adrenal (HPA) axis^41^. It is known that the PFC plays an important role in regulating HPA response to stress^42^. Therefore, exposure to excessive sGCs can affect the limbic and prefrontal regions, given the abundance of glucocorticoid receptors in these brain regions^43,44^. More specifically, children who were prenatally exposed to sGCs showed significant bilateral cortical thinning, particularly in the rostral anterior cingulate cortex (rACC)^45^. Given the critical role of the ACC in cognitive control and performance monitoring, the effect of prenatal exposure to sGCs on cortical thinning in this area may affect the development of performance monitoring. Indeed, a recent study from our group^46^ observed reduced behavioural response consistency and attenuated N2 amplitude during a Go/NoGo task in adolescents who had been prenatally exposed to sGC. Further source localization analyses of the EEG activity observed at the scalp level revealed that activations in the ACC and precuneus were also attenuated in adolescents who had been exposed to antenatal sGCs. These findings indicate that individual differences in the development of inhibitory control process triggered by the monitoring of stimulus–response conflict is associated with prenatal perturbation of the steroid hormones. However, it remains unclear as to whether the development of other performance monitoring processes, such as novelty and error monitoring, would also be associated with prenatal exposure to sGCs. Although the development of stimulus–response conflict and error monitoring have attracted much research attention in the past (see Ref.^37^ for review), studies that investigate novelty-related processes and directly compare the development of novelty and error monitoring are still scarce.

Therefore, the current study aims to compare the development of error and novelty monitoring in childhood and adolescence using a variant of the Flanker task that also includes a novelty manipulation component^24^. As an overview (see “Methods” section for details)^24^, during the Flanker part of the task, a string of 5 letters were horizontally presented on the screen. The letter shown in the centre was the target stimulus that was “flanked” by 2 letters on its left and right sides. The letters beside the target stimulus shown in the middle were the flankers. Participants were asked to respond to the target letter in the middle, which were either mapped onto the same or opposite responses as the flankers, thus resulting in compatible and incompatible trial combinations. In the novelty-oddball part of the task, participants either viewed a standard stimulus, a target stimulus, or a novel stimulus. Participants were instructed to respond when they detected the target and they were not instructed to respond upon the detection of a novel stimulus, as the presence of the rare novel stimuli were unbeknownst to the participants. The main advantage of this paradigm is that it allows one to study the processes underlying both error and novelty monitoring in the same task. This reduces any confounding effects due to different stimuli properties and thus can better disentangle the mechanisms underlying these two monitoring processes.

Based on previous findings from studies on the development of ERN^37^ and a study using the identical Flanker combined with novelty task in younger adults^24^, we hypothesized that the behavioural measures of error and novelty monitoring improve with age, but may show different time courses. Concomitant with performance improvement at the behavioural level, we also expected age-related effects on the neural correlates of error and novelty monitoring. Specifically, we expected that there will be stronger ERN activity but reduced N2 activity in adolescents as compared to children. Lastly, we also explored whether prenatal exposure to sGCs moderates the development of error and novelty monitoring. To investigate these questions, we conducted a cross-sectional study that included children and adolescents who were either exposed or not exposed to antenatal sGCs. Thus, the SGC group consisted of children and adolescents whose mothers experienced pregnancy complications with a serious risk of preterm delivery and received a single dose of SGC treatment (i.e. dexamethasone or betamethasone). The comparison group consisted of children and adolescents whose mothers had neither pregnancy complications nor given sGC treatment. Based on a previously observed long-term effect of such prenatal sGC exposure on behavioural and brain processes of stimulus–response conflict monitoring in a Go/NoGO task^46^, one might expect attenuated development of error and novelty monitoring in the sGC exposed group. However, it should be noted that processing requirements of error and novelty monitoring differ considerably from those of conflict monitoring and inhibition, thus it remains open as to whether long-term effects of prenatal sGC exposure may generalize across different types of cognitive monitoring.

## Results

### Sample characteristics

The characteristics of the current study samples are summarized in Table 1.

**Table 1:** Sample characteristics including demographic variables, perinatal variables and basic cognitive abilities.

Group	Children sample		*p-*value	Adolescents sample		*p-*value
	sGC (*n* = 21)	Controls (*n* = 32)		sGC (*n* = 28)	Controls (*n* = 24)	
**Demographic variables**						
Age	8.43 ± 1.16	8.72 ± 1.05	0.43	16.25 ± 1.21	16.42 ± 0.97	0.72
Sex (%male)	47.62	56.25	0.58	67.86	54.17	0.40
**Perinatal variables**						
Birth weight (g)	3260.95 ± 498.38	3307.40 ± 472.56	0.75	3080.00 ± 549.48	3357.08 ± 494.29	0.23
Birth length (cm)	50.62 ± 1.99	50.20 ± 2.48	0.49	49.57 ± 2.70	50.13 ± 2.19	0.47
Head	34.93 ± 1.71	34.38 ± 1.34	0.61	34.69 ± 1.29	34.82 ± 1.30	0.79
APGAR (5 min)	8.95 ± 0.92	8.88 ± 1.51	0.79	9.25 ± 0.65	9.50 ± 0.51	0.17
Length of gestation (w)	39.24 ± 1.48	39.55 ± 1.33	0.42	38.53 ± 1.26	39.24 ± 1.17	0.03*
**Basic cognitive abilities**						
IPT RT	3368.84 ± 762.22	3447.77 ± 665.01	0.56	2064.21 ± 295.59	2028.74 ± 252.19	0.64
SaW	2.70 ± 3.63	3.50 ± 2.98	0.15	13.21 ± 4.70	14.71 ± 5.86	0.48

Values are displayed in mean ± SD. APGAR is a measure of the health of the newborn that assesses Appearance, Pulse, Grimace, Activity and Respiration.

*IPT RT* reaction times in Identical Pictures Task, *SaW* correct reports in Spot-a-Word-Task. Where data is normally distributed, independent samples t-tests were conducted. Otherwise, Mann–Whitney U tests were conducted.

In both of the children and the adolescent samples, the sGC and comparison groups did not significantly differ in demographic variables (age and gender distribution, all *p* > 0.05), perinatal variables (birth weight, birth length, head circumference and APGAR score, all *p* > 0.05), or basic cognitive abilities (perceptual speed processing, verbal knowledge, all *p* > 0.05). In the adolescent sample, participants in the sGC group were born approximately one week earlier compared to the comparison group (p = 0.03). As such, the statistical models for the main results of the study were repeated with length of gestation as a covariate. However, this did not change the results and is thus not reported. We also examined potential age differences in demographic and perinatal variables between children and adolescents separately for the comparison and the SGC groups. The only significant difference was observed in the comparison group for the APGAR score, with adolescents showing a higher score (p = 0.02) than children. However, controlling for this variable did not affect the pattern of observed effects.

Due to technical issues during data collection, measures assessed with the experimental task were missing in some participants, thus the final sizes of the subsamples for the analyses reported below varied slightly between the analyses. Specifically, the following numbers of participants could not be included for the relevant analyses due to missing measures: 1 child in the sGC group did not have the PES data, and 1 adolescent in the sGC did not have task data. Since it is well established that performance accuracy and response speed improves with increasing age during development, we also conducted analyses that control for overall performance accuracy or reaction times for the key measures associated with novelty or error monitoring at the behavioural and brain levels. The vast majority of results did not differ between models without or with these two variables as covariates. Thus, we only report results from models without including accuracy or reaction times as a covariate, but only report two specific effects where a difference was observed at the behavioural level.

### Behavioural results

#### Flanker task performance

Overall, adolescents demonstrated better performance than children, with faster reaction times, *F*(1,101) = 32.16, *p* < 0.001, lower reaction time variability, *F*(1,101) = 110.29, *p* < 0.001, higher accuracy rate, *F*(1,101) = 69.79, *p* < 0.001, and correspondingly, lower error rate, *F*(1,101) = 49.62, *p* < 0.001. There was neither significant difference between the sGC and comparison groups, nor significant Age × Group interactions across all performance measures (all *p* > 0.29).

#### Behavioural adaptation to novel stimuli or errors

**Figure 1:**
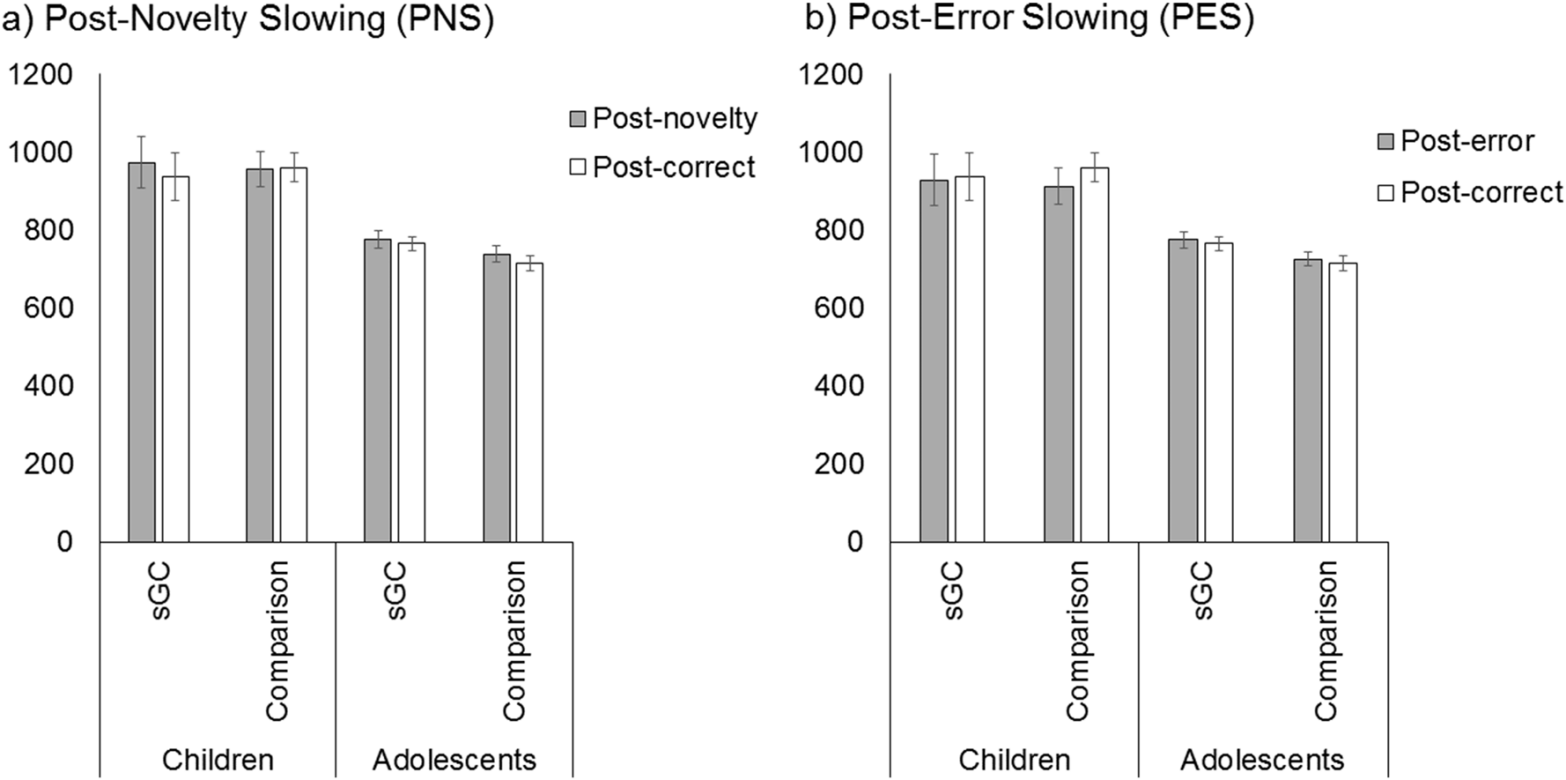
Reaction times for (**a**) post-novelty trials and (**b**) post-error trials. Note that in both cases, performance is comparable across sGC and comparison groups. Error bars represent ± 1 standard error of mean.

To test potential age and group effects on behavioural adaption to novelty or response error, reaction times after different trial types were further analysed. For post-novelty slowing, there was a significant main effect of Age, *F*(1,100) = 29.63, *p* < 0.001 indicating that adolescents responded faster than children. Of note, the main effect of Trial was also significant, *F*(1,99) = 6.36, *p* = 0.01, with post-novelty trials being slower than post-correct trials (Fig. 1a) No other main effects or interactions were statistically significant (all *p* > 0.05). If overall RT was included as a covariate in the model, we also observed a significant main effect of group, *F*(1,99) = 11.83, *p* < 0.001, with the comparison group showed a greater post-novelty slowing as compared to the sGC group.

As for post-error slowing, there was a significant main effect of Age, *F*(1,100) = 25.04, *p* < 0.001, again demonstrating that adolescents responded faster than children. The main effect of Trial in this case was not significant. However, there was a significant Age × Trial interaction, *F*(1,98) = 8.31, *p* = 0.005 (see Fig. 1b). Post-hoc tests revealed that children were slower on post-correct than post-error (*p* = 0.033), while adolescents were slower on post-error than post-correct (*p* = 0.04). Note that this effect in adolescents was not significant after correcting for multiple comparisons (*α* = 0.025). No other main effects or interactions were statistically significant (all *p* > 0.08). If overall RT was included as a covariate in the model, we also observed a significant main effect of group, *F*(1,99) = 10.17, *p* = 0.002, with the comparison group showing a greater post-error slowing as compared to the sGC group.

### ERP analyses

#### The N2 component

**Figure 2:**
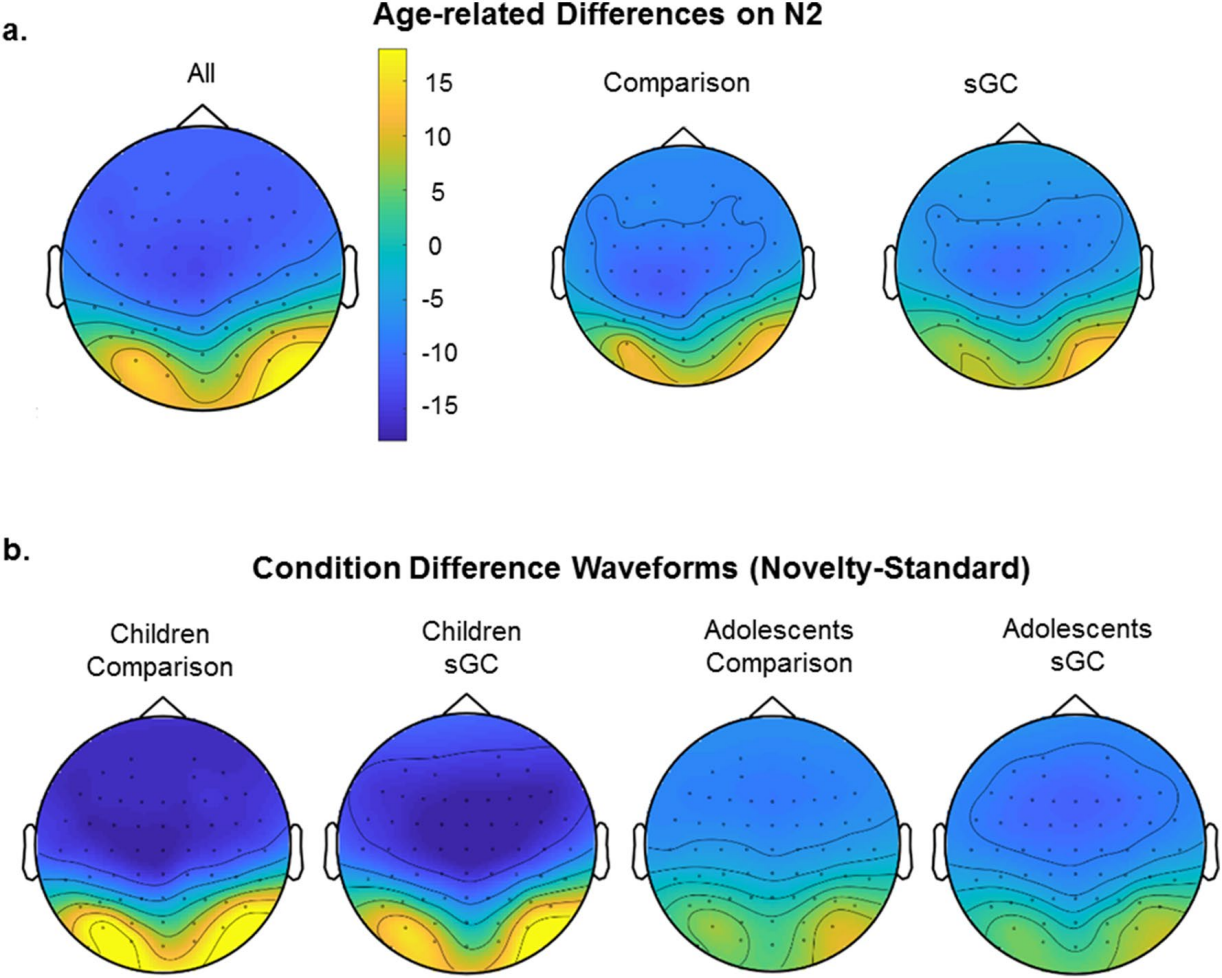
(**a**) Novelty-related N2 amplitudes showed maximal age-related difference at the midline electrodes (in particular, electrode Cz) between 200 to 600 ms after the onset of stimulus; (**b**) topographical maps showing maximal condition difference for children in the comparison group, children in the sGC group, adolescents in the comparison group, and adolescents in the sGC group at peak latency (± 25 ms).

**Figure 3:**
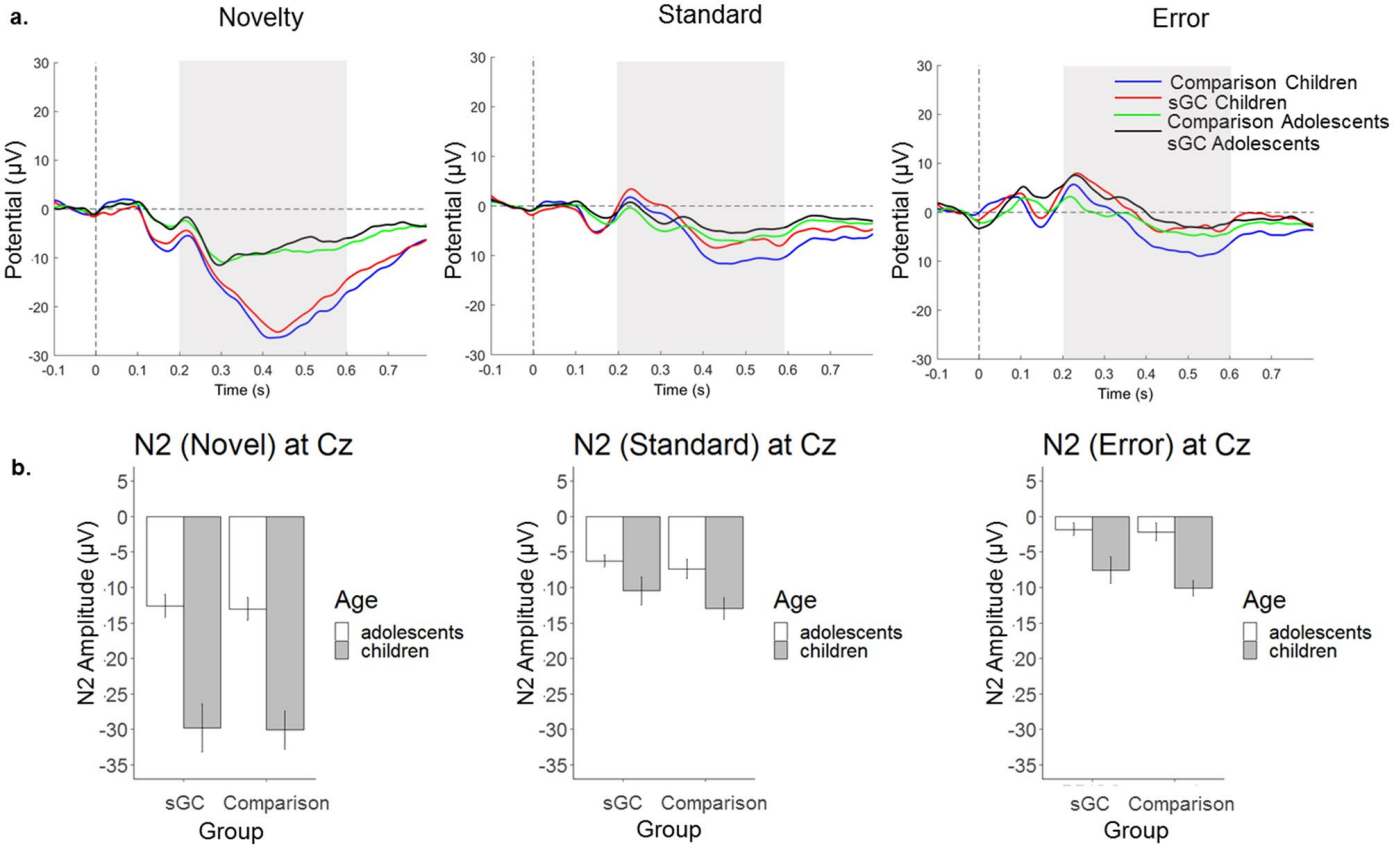
(**a**) Grand averaged ERP waveforms at Cz for Novel, Standard and Error stimuli; (**b**) Interaction between Age and Stimuli on N2 amplitude at Cz (Error bars represent ± 1 standard error of mean);

N2 was quantified at electrode Cz where there was maximal difference between age groups (see Fig. 2a). This is consistent with previous studies which found that novelty-related activity such as N2 was largest at central midline (Cz^24,47–49^). For separate subgroup topographical maps and grand averaged ERP waveforms, see Figs. 2b and 3a. There was a significant main effect of Age, *F*(1,101) = 38.53, *p* < 0.001, showing a stronger N2 in children as compared to adolescents, and a significant main effect of Trial (type), *F*(2, 200) = 245.96, *p* < 0.001, where a stronger N2 was observed for Novel than Error, *p* < 0.001 and Standard, *p* < 0.001. Follow-up tests showed that N2 in Standard trials was also larger than in Error trials, *p* < 0.001. In terms of interaction, there was a significant Age x Trial interaction, *F*(2, 200) = 36.62, *p* < 0.001 (see Fig. 3b). In both age groups, Novel stimuli elicited stronger N2 than Error (children, *p* < 0.001; adolescents, *p* < 0.001) and Standard (children, *p* < 0.001; adolescents, *p* < 0.001). A stronger N2 was also observed for Standard than Error trials in both age groups (children, *p* = 0.002, adolescents, *p* < 0.001). Furthermore, children also displayed a stronger N2 than adolescents across all types of stimuli (novel, *p* < 0.001; standard, *p* < 0.001; error, *p* < 0.001). No other effects and interactions were statistically significant (all *p* > 0.40).

#### The ERN component

**Figure 4:**
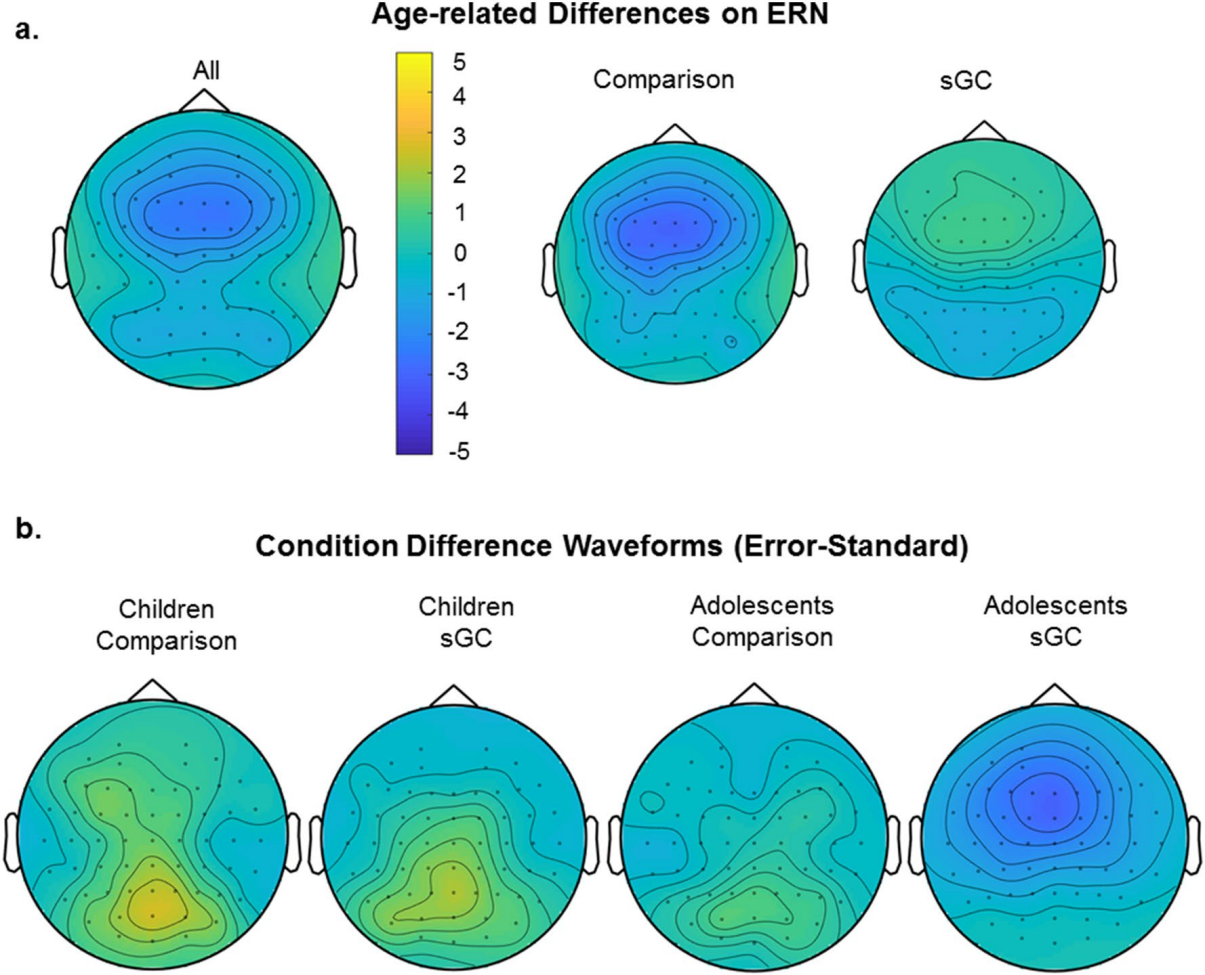
(**a**) Error-related ERN amplitudes showed maximal age-related difference at the fronto-central electrodes (in particular, electrode FCz) between 50 ms prior to 100 ms after the onset of stimulus; Topographical maps showing maximal condition difference for children in the comparison group, children in the sGC group, adolescents in the comparison group and adolescents in the sGC group at peak latency (± 25 ms).

**Figure 5:**
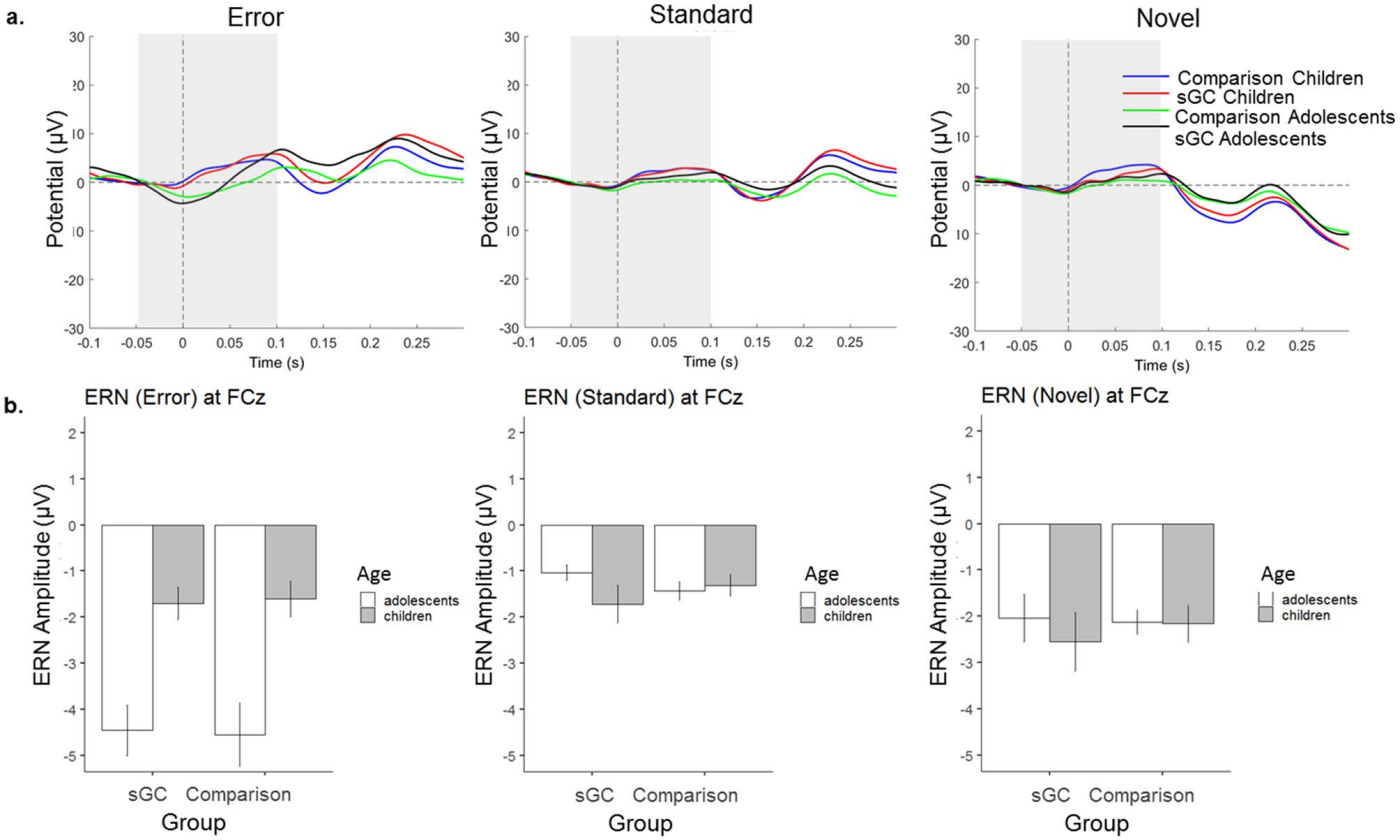
(**a**) Grand averaged ERP waveforms at FCz for Novel, Standard and Error stimuli, (**b**) Interaction between Age and Stimuli on ERN amplitude (Error bars represent ± 1 standard error of mean).

ERN was quantified at electrode FCz where there was a maximal difference between age groups (see Fig. 4a), consistent with previous studies which demonstrated that error-related ERN is maximal and most reliable at FCz^50–53^. For separate subgroup topographical maps and grand averaged ERP waveforms, see Figs. 4b and 5a. Given that there is a strong positive peak at the posterior site, we also analyzed ERN at Pz which yielded similar results to FCz (see Supplementary Information). In line with previous studies, we only presented the results from FCz here. There was a significant main effect of Age, *F*(1,100) = 6.73, *p* = 0.01, where adolescents showed stronger ERN than children and a significant main effect of Trial (type), *F*(2,197) = 20.62, *p* < 0.001, where Errors elicited larger ERN than Novel stimuli (*p* = 0.01) and Standard stimuli (*p* < 0.001) and novel stimuli elicited stronger ERN than standard stimuli (*p* < 0.001). There was also a significant Age x Trial interaction, *F*(2,197) = 24.30, *p* < 0.001 (see Fig. 5b). Post-hoc tests revealed that Errors elicited stronger ERN than Novel (p < 0.001) and Standard (*p* < 0.001) only in adolescents but not in children. Furthermore, adolescents displayed a stronger ERN than children only during Errors (*p* < 0.001) but not during other trial types. No other main effects or interaction were statistically significant (all *p* > 0.45).

For all measures, gestational length was not a significant covariate (*p*s > 0.26). Even after controlling for gestational length, the results of the brain and behaviour differences between both children and adolescents remain the same.

#### Brain-behaviour correlational analyses of error and novelty monitoring

**Figure 6:**
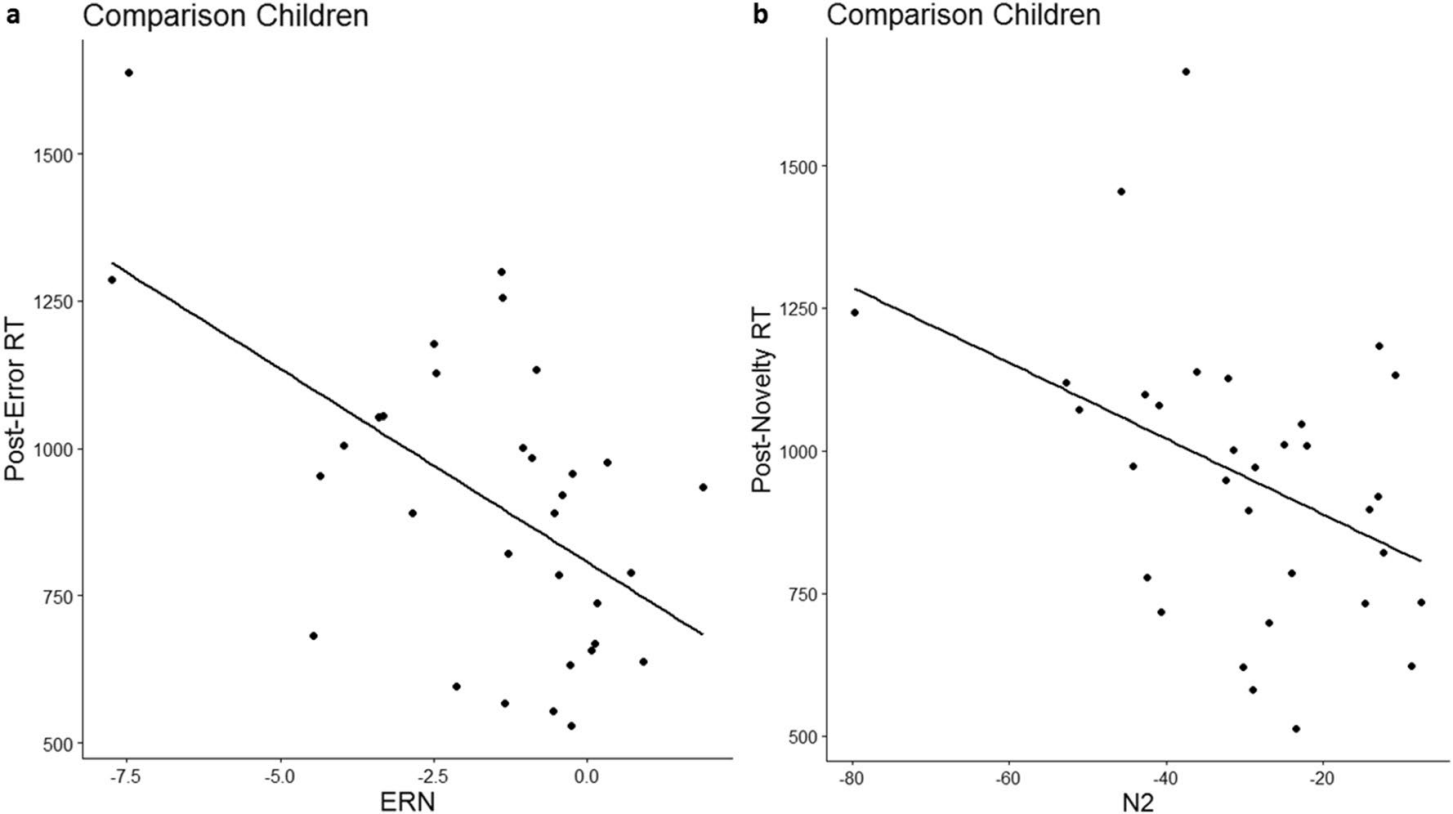
(**a**) Stronger ERN was significantly correlated with increased post-error reaction times (increased PES); (**b**) Stronger N2 (more negative N2 amplitude) was marginally associated with increased post-novelty reaction times (increased PNS) for children in the comparison group.

Regarding error monitoring, we found that stronger ERN was significantly correlated with increased post-error reaction times (increased PES) for children in the comparison group, *rho* = − 0.50, *p* = 0.004 (see Fig. 6a) but not for children in the sGC group, *rh*o = − 0.37, *p* = 0.108. There was no significant difference in the correlation coefficients, p = 0.30 (one-tailed, p = 0.60 for two-tailed test). There was no significant correlation between post-error reaction times and ERN for adolescents in the comparison (*rho* = − 0.07, *p* = 0.74) or in the sGC group (*rho* = 0.29, *p* = 0.16). For novelty monitoring, we observed that stronger N2 (more negative N2 amplitude) was marginally associated with increased post-novelty reaction times (increased PNS) for children in the comparison group, *rho* = − 0.39, *p* = 0.026 (see Fig. 6b) but not in the sGC group, *rho* = 0.025, *p* = 0.92. A marginally difference in the correlation coefficients between these two groups was observed, p = 0.076 (one-tailed, p = 0.15, two-tailed). There was no significant correlation between N2 and post-error slowing for adolescents in the comparison group, (*rho* = 0.34, *p* = 0.11) and in sGC group (*rho* = 0.28, *p* = 0.17).

## Discussion

The present study extends previous research on performance monitoring by investigating the development of error and novelty monitoring and their corresponding neural correlates (ERN and N2, respectively) across childhood and adolescence. We also explored potential effects of prenatal sGC exposure on the behavioural and brain indicators of error and novelty monitoring. By using a combined flanker with novelty monitoring task^24^, as anticipated, we found that adolescents displayed better overall behavioural performance as shown by higher accuracy rate, lower error rate, faster reaction times and lower reaction time variability. These general findings are in line with previous evidence indicating that the maturation of cognitive control skills, specifically performance monitoring which implicates the maturation of the frontal network^37,54^, protracts into adolescence. Furthermore, as expected, the results showed age-related differences in electrophysiological correlates of error and novelty monitoring measured from fronto-central electrodes. Whereas the amplitude of error-related ERN- is larger in adolescents than in children, the amplitude of novelty-related N2 is larger in children than in adolescents. Although previous evidence lends support to a common underlying neural substrate for both types of performance monitoring^24^, the different requirements of these two monitoring processes may nonetheless contribute to different patterns of age differences in error and novelty monitoring at the behavioural and brain levels. In the following subsections, we discuss these developmental differences in detail.

To date, studies using other conflict paradigms (e.g., conventional Flanker task, Go/Nogo task) did not demonstrate obvious developmental effects of PES at the behavioural level despite demonstrating developmental effects on the ERN^33,55,56^. In the current study using the flanker combined with novelty monitoring task, we observed a significant age by trial (error vs. correct) interaction on reaction time. Results from post-hoc tests, corrected for multiple comparisons, revealed trends that indicate PES was only observed in adolescence but not in children who actually reacted quicker on post-error than post-correct trials. Using the same task, Wessel et al.^24^ previously found evidence for error-related behaviour adaptation (i.e., significant PES effect) in younger adults. Together, these findings suggest that the task used in this study and in Wessel et al.^24^ study is sensitive to behavioural developmental effects and reveal less developed error-related behavioural adaption, as captured by PES, in childhood compared to adolescence or adulthood.

As for novelty-related behavioural adaptation, we observed an overall increase in reaction times for post-novelty trials relative to post-correct trials, indicating general post-novelty slowing. However, there was no interaction with age, demonstrating that there is no difference between children and adolescents with regards to post-novelty slowing. These findings indicate that, unlike error-related behavioural adaption which only reach sufficient maturation in adolescence, novelty-related behavioural adaption is already developed in childhood. The different developmental time courses of these two processes may reflect differences in the processing requirements of error and novelty monitoring. Being able to monitor task-relevant information as in the case of one’s own performance errors without external feedback, relies more on endogenous top-down control which is neurocognitively demanding. On the other hand, being able to monitor salient task-irrelevant external stimuli as in the case of novelty monitoring, can, in part be triggered by bottom-up stimulus-related processes. This explanation is in line with the recently proposed adaptive orienting theory of error processing^57^, which stipulates that errors and novel events share automatic processing, but that errors invoke additional, more strategic processes. The results of the current study suggest that these later processes only emerge later in development. These age-related differences in error and novelty monitoring performance, when taken into consideration with differences in the directions of age effects on their corresponding neural correlates, further shed light on the functional roles of ERN and N2.

Our findings with regards to developmental effects on the neural correlates of error and novelty monitoring is consistent with previous studies^31,34–36,38^, with ERN increasing with age and N2 decreasing with age. Specifically, whereas adolescents displayed a stronger fronto-central ERN at FCz as compared to children, children displayed a stronger central maximal N2 at Cz as compared to adolescents. The ERN has been established as part of a neural error processing system that helps to regulate actions and learning which is reflected in post-error adjustments of behaviour such as post-error slowing (PES). In the current study, we observed post-error slowing only in adolescents, who as a group, also showed a stronger ERN than children. Furthermore, despite an overall weaker ERN in children, individual differences in the amplitude of ERN correlated with behavioural performance in children: a larger ERN is significantly associated with greater PES among children in the comparison group. Together, the direction of age-related difference and the observed brain-behaviour relation underscore that the amplitude of ERN reflects the extent of neural processes recruited for behavioural adjustments to regulate actions following errors, and these processes are less developed in children. Although PES has been thought to either represent compensatory behaviour to improve chances for accurate responding in subsequent trials^58^ or represent an orienting response caused by an error^57^, a direct correlation between PES and post-error accuracy is not always observed^16^. We also did not observe a direct correlation at the behavioural level, but observed a correlation between the amplitude of ERN and post-error accuracy in the comparison group (rho = − 0.51, p < 0.001), albeit this relation is in part shared with the effect of age. Thus, the PES and ERN may not only signal the needs of top-down control, but also reflect processes associated with unanticipated process disruption due to errors instead of an adaptive signal^14–16^.

Contrary to ERN, we showed that the amplitude of novelty-related N2 decreases with age. This finding is in line with results from previous studies on developmental effects in response conflict related N2 (see Ref.^37^ for review). Furthermore, consistent with previous results observed in younger adults using the same task^24^, the amplitude of N2 was stronger for novel stimuli as compared to standard and error stimuli and this effect was observed in both children and adolescents. The fronto-central N2 is usually elicited during the occurrence of novel and unfamiliar stimuli, as demonstrated by oddball paradigms^59^. Since both children and adolescents demonstrate post-novelty slowing, the greater novelty-associated N2 amplitude observed in children relative to adolescents could suggest that neural processes in children are relatively more susceptible to bottom-up task-irrelevant stimulus triggered processes. When children are engaged in novelty monitoring processes, they recruit more activity in the fronto-central network for novelty-related behavioural adaption. Indeed, for children in the comparison group, greater post-novelty slowing at the behavioural level was significantly associated with a stronger (i.e., more negative) N2.

Previously, it has been shown that behavioural response consistency and N2 were reduced in adolescents exposed to antenatal SGC on stimulus–response conflict monitoring assessed with a Go/No-go task^60^. Contrary to these findings, the current findings did not demonstrate any effects of antenatal SGC on error and novelty monitoring in children or in adolescents. This discrepancy in findings could be attributed to the different types of cognitive monitoring processes tapped by different tasks. For instance, the Go/No-go task taps into processes of top-down inhibition and stimulus–response conflict monitoring. Although error monitoring also requires exogenous control, no response inhibition is required; this differs from the Go/No-go task. The monitoring of salient novel stimuli in the Flanker combined with novelty monitoring task is less dependent on endogenous cognitive control. Earlier studies found that attentional deficits following prenatal stress were only manifested when endogenous control was required while externally triggered exogenous control was less affected^61,62^. Therefore, while antenatal sGCs has a prominent effect on endogenous cognitive control, it has lesser effect on exogenous cognitive control. Although there was no direct effect of antenatal sGC exposure on the development of error and novelty monitoring at the behavioural or brain level, the relations between PES or PNS and their respective electrophysiological correlates might be altered by the prenatal hormonal perturbation. Unlike children in the comparison group, the link between the ERN or novelty-related N2 and behavioural adaption could not be observed in children prenatally exposed to sGC, suggesting that prenatal perturbation of steroid hormones may alter the brain-behaviour relations. However, we highlight the tentative nature of this finding, given that only a marginal difference was observed when directly comparing the difference in the strengths of the correlations between the comparison and sGC groups. Nevertheless, this initial result is in line with the finding from an earlier study^45^, which showed that fetal glucocorticoid exposure alters the relation between the thickness of ACC and affective behaviour in children, with the effect only observed in the comparison group but not in the group exposed to sGCs. Findings from another recent study may also help to interpret the group difference in the brain-behaviour relations. It has been recently shown that perturbations in prenatal hormonal conditions (e.g., due to elevated maternal cortisol level) are associated with brain functional reorganization that is expressed as altered brain network connectivity^63^. The extent of such alterations may differ substantially between the prenatally exposed individuals, thus rendering “normative” brain-behaviour relations to be less consistent in this group. Future research are needed to systematically investigate the potential effects and mechanisms of prenatal hormonal influences on altering brain-behavioural relations.

In summary, the current study demonstrated age-related differences in neural mechanisms underlying error and novelty monitoring between children and adolescents. Furthermore, the developmental time courses differ between these two monitoring processes. Error-related behavioural adaption shows a more protracted development than novelty monitoring. Although only adolescents showed post-error slowing, post-novelty slowing was observed both in children and adolescents. Whereas immature error monitoring processes in children are reflected in the weaker amplitude of ERN compared to adolescents, the larger novelty-related N2 in children may reflect their susceptibility to salient task-irrelevant stimuli and over-recruitment of the underlying neural processes. Neither monitoring process seems to be affected by prenatal perturbation of the glucocorticoid system. However, this prenatal alteration of steroid hormones altered the normative brain-behaviour relations. Future studies need to examine the potential effects of prenatal sGC exposure on the reorganization of brain connectivity to gain a mechanistic understanding. Results from the current cross-sectional study could be susceptible to other factors contributing to individual and cohort differences, thus, longitudinal studies should be pursued in the future to further enlighten the developmental effects on error and novelty monitoring processes.

## Materials and methods

### Participants

The data analysed in the current study comprised of an adolescent sample (14–18 years old; *n* = 52) and a children sample (7–12 years old; *n* = 53). The adolescent sample was re-recruited from previous studies on long-term impacts of prenatal sGC on cortisol release during social stress^64^ and intelligence^65^ in childhood. The children sample was newly recruited in cooperation with the Department of Gynaecology and Obstetrics at the Medical Faculty of Technische Universität Dresden. The inclusion criteria of potential participants were children who were term-born (≥ 37 weeks of gestation) and not exposed to paediatric intensive care (see Table 1 in Result section for details of sample characteristics^46^). From these two samples, behavioural and EEG data collected during a Go/NoGo task in the adolescent sample have been reported in a previous study^46^. Hair steroid levels as a marker of integrated long-term HPA-axis activity in the children and adolescents were reported in another prior study^60^. However, the behavioural and EEG data collected during a combined Flanker with novelty manipulation task^24^ in both samples and the performances of error and novelty monitoring performances along with their EEG correlates investigated in the current study have not been reported before.

### Ethics and informed consent

Informed consent from both custodians of children and adolescents were obtained prior to their study participation. The current study was approved by the local ethics committee of the TU Dresden (EK 235062014) and conducted according to the principles of the Declaration of Helsinki.

### Subgroups defined by age and status of prenatal sGC exposure

Both adolescent and children samples further consisted of two subgroups. The first group is the sGC group, which included children and adolescents of mothers who had experienced pregnancy complications with serious risk of preterm delivery (i.e., vaginal bleeding, cervical insufficiency, premature labour pain) and received the common sGC therapy to accelerate fetal lung maturation. The antenatal sGC treatment consisted of either dexamethasone (DEX) or betamethasone (BETA), administered respectively in four doses of 6 mg every 12 h or in two doses of 12 mg every 24 h. The second group is the comparison group which included children and adolescents of mothers who had neither experienced pregnancy complications nor had been given sGC treatment.

#### Adolescent sample

The characteristics of this sample were described in detail previously^46,60,64,65^. Briefly, obstetrical documents and paediatric examination booklets of all mothers who delivered their babies between 1997 and 2003 were screened. All potential participants belonging to the sGC group (*n* = 304) and the comparison group (*n* = 372) were invited to participate in the study. A total of 101 participants in the sGC group and 96 participants in the comparison group agreed to participate in the previous studies in their childhood^64,65^. Out of these participants, 52 adolescents (14–18 years; 28 sGC group, 24 comparison group) participated in the current study that also included EEG assessments of performance monitoring.

#### Children sample

Out of 8421 individuals who met the inclusion criteria (see above), obstetrical documents from all mothers who delivered their babies between 2005 and 2010 at the Clinic of Gynaecology and Obstetrics, University Hospital Carl Gustav Carus were screened by medical staff. Invitations for study participation were sent to parents of all children who were prenatally exposed to sGC (*n* = 523) and parents of all children in the comparison group (*n* = 502). Out of all invited potential participants, a total of 63 children and their parents agreed to participate in the study. Data from 10 children had to be excluded from the current analyses (3 comparison group; 7 sGC group) because they did not respond as instructed and/or did not complete the EEG assessments. Thus, the final effective sample included 53 children (7–12 years; *n* = 21 in the sGC group and *n* = 32 in the comparison group).

### Demographic variables and basic cognitive measures

Approximately 2 weeks before performing the experimental task, parents and their children were asked to fill in a set of questionnaires to assess demographic characteristics (age, sex) and birth-related characteristics, including length of gestation, birth weight, birth length, head circumference, APGAR (Appearance, Pulse, Grimace, Activity and Respiration) score assessed 5 min after birth. Participants’ basic cognitive abilities were also measured, where perceptual speed processing was assessed with the Identical Pictures Task and verbal knowledge was assessed with the Spot-a-Word task^66^.

### Experimental paradigm

To investigate developmental differences in novelty and error monitoring, we used a combined flanker with novelty monitoring task^24^. This task consists of two parts, i.e., the flanker part and the novelty-oddball part. The Flanker part of the task consisted of a letter version of the Eriksen flanker task^67^ where several letters (H, Z, S, X) were mapped to each side. For instance, H and Z were mapped to the left side while S and X mapped to the right side, with the mapping counter-balanced across participants. Stimulus–response mapping was displayed on the screen throughout the experiment. The stimuli included a five-letter stream with the middle element as the “imperative stimulus” while the left and right elements served as flankers, resulting in compatible (imperative stimulus and flankers mapped to the same side, e.g., HHZHH) and incompatible (imperative stimulus and flankers mapped to opposite sides, e.g., HHXHH) trial combinations. Each trial started with a fixation period that was pseudo-randomly jittered (0, 400, 700, 900, 1100, or 1500 ms), followed by the stimulus which was presented for 70 ms. Participants were instructed to press ‘1’ for target letters mapped to the left and ‘2’ for target letters mapped to the right within an adaptive response time window.

The novelty-oddball part started ten milliseconds after participants responded in the flanker part of the task. During the novelty-oddball part of the task, one of three kinds of stimuli were presented for 400 ms; a standard stimulus (upward-pointing triangle), a target stimulus (downward-pointing triangle) or a novel stimulus which was a drawing of everyday objects taken from the International Picture Naming Project database^68,69^. Participants were instructed to monitor the triangles and press a third button whenever they detected the target (downward-pointing triangle) while the novel stimuli were uninstructed and did not require any form of response. The standard stimulus (upward-pointing triangle) appeared after both correct and error responses during the flanker part of the task, whereas the target triangle (downward-pointing triangle) appeared after three correct responses that were spread across the experiment. The novel stimulus appeared only after correct responses, with the quantity of novel stimuli individually matched to the number of errors in the flanker part of the experiment (see Wessel et al.^24^ for other technical details of the paradigm and algorithm).

### EEG recording

Participants’ EEG data were recorded in an acoustically and electrically shielded chamber while participants performed the combined flanker with novelty monitoring task. EEG activity was recorded using 64 active Ag/Al electrodes, positioned according to the 10/20 system using Brain Vision Recorder (BrainAmp DC amplifiers, Brain Products GmbH, Gilching, Germany) with a sampling rate of 500 Hz. The reference electrode was placed at the left mastoid while the ground electrode was placed at position AFz. Electrodes for horizontal and vertical electro-oculograms were placed at the outer canthi of the eyes and below the right eye, respectively. Impedances were kept below 10 kΩ.

### EEG pre-processing

EEG data was re-referenced offline to the averaged mastoids using Brain Vision Analyzer. After re-referencing, the following pre-processing steps were performed using the open-source EEGLAB toolbox^70^ and Fieldtrip toolbox^71^ for MATLAB. EEG data was down-sampled to 250 Hz and bandpass-filtered in the range of 0.5–30 Hz. Subsequently, the data were epoched into stimulus-locked segments from 500 ms before to 2500 ms with respect to stimulus onset. Epochs with severe muscular artifacts were manually rejected and Independent Component Analysis (ICA) was applied to remove components of ocular and muscular artifacts. Then, the data was segmented in a response-locked manner, with a time window of 100 ms before and 900 ms with respect to the response in order to create epoched segments corresponding to the erroneous, standard and target stimuli. The resulting epochs were then baseline corrected with the time window preceding the stimulus from 100 ms before to stimulus onset (-100 ms to 0 ms) serving as baseline. Participants were excluded for analyses if there were less than 2 epochs remaining for each condition^72^.

### ERP quantification and analyses

The trial epochs were averaged separately for each trial type (Standard, Novel, Error) and each participant to extract the relevant ERP components. Peak amplitude for the N2 component was defined as the most negative amplitude in the time window of 200–600 ms for children and 200–450 ms for adolescents after stimulus onset, separately for the standard, error and novel trials based on the grand average waveforms. Peak amplitude for the ERN component was defined as the most negative peak in the time window between – 50 to 100 ms relative to error onset. Individual mean amplitudes for each component were obtained ± 25 ms around the individual peak for each participant.

### Statistical analyses

Statistical analyses were performed using the RStudio (Version 1.3.959, The R Foundation of Statistical Computing, www.rstudio.com). Outliers below or above 3 standard deviations of the mean were excluded from the main analyses. For all statistical tests, the *α* level was set at 0.05 and adjusted using Bonferroni correction in case of multiple comparisons.

Behavioural performance and ERP mean amplitudes were analysed with linear mixed-effect models using maximum-likelihood estimation with participants as a random intercept. This analysis was applied using the lme function of the nlme package in RStudio. In order to test for potential group and developmental differences in behavioural performance, linear mixed-effects models were conducted with group (sGC, comparison) and age (children, adolescents) as between-group fixed factors for each behavioural performance measure (accuracy rate, error rate, reaction time and reaction time variability).

Behavioural adaptations to novel stimuli were analysed using linear mixed-effects model with group (sGC, comparison) and age (children, adolescents) as between-group fixed factors and trial type (post-novel, post-correct) as the within-group factor. Similarly, behavioural adaptations to errors were analysed using linear mixed-effects model with group and age as between-group fixed factors and trial type (post-error, post-correct) as the within-group factor. Similar linear mixed-effects models were conducted for each ERP component (N2, ERN) with group (sGC, comparison) and age (children, adolescents) as between-group factors, and trial type (novel, standard, error) as within-groups factor.

To explore the brain-behaviour relations during cognitive monitoring, we conducted explorative correlational analyses using Spearman’s rho to investigate the following relations: (1) Brain-behaviour association of error monitoring; (2) Brain-behaviour association of novelty monitoring; For each of these correlational analyses, the correlational analyses were performed separately for the age (children vs. adolescents) by sGC exposure group (sGC vs. comparison) combinations; therefore, the *α* level (0.05) was divided by the number of correlational analyses conducted (*n* = 4), with family-wise corrected α value for each for each of these six relations being set at *α* = 0.0125. Fischer *r*-to-*z* test was used to analyse the difference in correlation coefficients between groups.

## Supplementary Information

Supplementary Information.

## Data Availability

The anonymised behavioural and EEG data will be made available for research purposes upon request.
