# Supplementary material for: Neurocognitive development of novelty and error monitoring in children and adolescents
Source: Sci Rep. 2021 Oct 6;11:19844. doi: 10.1038/s41598-021-99043-z (PMC8494897; doi:10.1038/s41598-021-99043-z)
Supplement: Supplementary file 1 — Supplementary Information. [file 41598_2021_99043_MOESM1_ESM.docx]

**Supplementary Materials**

The ERN Component at Pz

There was a significant main effect of Age, *F*(1, 100)=6.34, *p*=0.01 where children showed a more positive peak (i.e., weaker ERN) than adolescents. There was a significant main effect of Trial, *F*(2, 198)=8.68, *p*=0.0002 where novel stimuli and standard stimuli showed a more positive amplitude than error stimuli (*p*=0.05, *p*<0.001 respectively). Standard stimuli also showed a more positive amplitude than Novel stimuli, p=0.03. There was also a significant Age x Trial interaction, *F*(2, 198)=3.10, *p*=0.047. Children showed a more positive amplitude than adolescents, only for errors, p=0.001. In adolescents only, Pz amplitude differentiated between the different types of Trial, where novel stimuli and standard stimuli showed a more positive amplitude than errors (*p*=0.003, *p*<0.001 respectively) and standard stimuli showed a more positive amplitude than novel stimuli, *p*=0.02. But, these differences were not observed in children. All other main effects and interactions were not statistically significant (all *p* > 0.25).

Brain-behaviour correlation with ERN at Pz

For children in the comparison group, a stronger ERN at Pz was associated with greater post-error slowing, *rho*=-0.45*, p*=0.012 but this was not observed for children in the sGC group, *rho*=-0.11, *p*=0.64. There was no significant difference in the correlation coefficients between these two groups, *p*=0.11 (one-tailed, *p*=0.22, two-tailed). There was no significant correlation between post-error slowing and ERN for adolescents in the comparison group, *rho*=-0.06, *p*=0.77 and sGC group, *rho*=0.06, *p*=0.79.
